# Perceptual decision-making in autism as assessed by “spot the difference” visual cognition tasks

**DOI:** 10.1038/s41598-022-19640-4

**Published:** 2022-09-14

**Authors:** Nazia Jassim, Adrian M. Owen, Paula Smith, John Suckling, Rebecca P. Lawson, Simon Baron-Cohen, Owen Parsons

**Affiliations:** 1 Autism Research Centre, Department of Psychiatry, University of Cambridge, Cambridge, UK; 2 Department of Psychiatry, School of Clinical Medicine, University of Cambridge, Cambridge, UK; 3 Department of Physiology and Pharmacology, The Western Institute for Neuroscience, London, Canada; 4 Department of Psychology, University of Western Ontario, London, Canada; 5 Department of Psychology, University of Cambridge, Cambridge, UK

**Keywords:** Psychology, Human behaviour, Cognitive neuroscience, Sensory processing, Visual system

## Abstract

Discriminating between similar figures proves to be a remarkably demanding task due to the limited capacity of our visual cognitive processes. Here we examine how perceptual inference and decision-making are modulated by differences arising from neurodiversity. A large sample of autistic (n = 140) and typical (n = 147) participants completed two forced choice similarity judgement tasks online. Each task consisted of “match” (identical figures) and “mismatch” (subtle differences between figures) conditions. Signal detection theory analyses indicated a response bias by the autism group during conditions of uncertainty. More specifically, autistic participants were more likely to choose the “mismatch” option, thus leading to more hits on the “mismatch” condition, but also more false alarms on the “match” condition. These results suggest differences in response strategies during perceptual decision-making in autism.

## Introduction

What makes “spot the difference” puzzles so challenging and why are some people better at these puzzles than others? The deceptively simple task of identifying the differences between two similar visual scenes highlights the complexity of human visual cognition^1^.

Actively discriminating between two similar images engages a cascade of steps from low-level processing of stimulus features to high-level object recognition. At the perceptual level, exposure to an object may generate expectations of similar, contextually-related objects^2,3^. For example, consider a scenario in which a person is asked to visually inspect two slightly different images, image A and image B, and decide whether they match or not. The more subtle the differences between the two images, the more uncertain the brain may be about the “true” state of the environment. After the initial visual processing,^3^ the overall “discriminability” of features in image A may lead to an expectation violation in image B, thus facilitating a perceptual decision^4–6^. However, what if image B is identical to image A? In this scenario, the lack of discernable differences may contribute to internal noise during perceptual inference leading to conflict or uncertainty during the decision process^7–9^. While performance on such tasks may boil down to inter-individual differences across various factors such as motivation, working memory, fluid intelligence, and visual attention^10–12^, it may also be modulated by differences in perceptual inference and decision-making as seen in autism spectrum conditions^13–16^.

In this article, we use the preferred identity-first language to describe people on the autism spectrum^17^. Autism spectrum conditions (henceforth autism) are a set of neurodevelopmental conditions characterized by difficulties in communication and relationships, alongside unusually narrow interests, repetitive, restricted patterns of behaviour, and sensory-perceptual differences^18^. Visual cognition is a prominent area of interest in autism research. Autistic people have been described as not “seeing the wood for the trees” due to their more “veridical” perception^19–22^. For example, autistic individuals have been found to consistently outperform typical participants in identifying hidden figures in complex scenes and in classic visual search paradigms^23,24^. However, it is important to note that autistic individuals have been found to be *faster*, but not necessarily more accurate in these tasks^23,25–28^. It is unclear how autistic participants make two-alternative perceptual decisions in such target detection tasks, particularly in trials where there is no target or “signal” present.

Optimal performance on a perceptual decision task requires filtering out of external noise and a reduction in internal noise^29–33^. While external noise encompasses environmental factors, such as task-relevant or task-irrelevant distractors, internal noise refers to variability in neuronal signals or random neuronal fluctuations that pose a challenge during perceptual inference and decision-making^34^. Neural models of autism suggest that, due to a possible imbalance of excitatory and inhibitory neurotransmitters, variable levels of internal noise may contribute to the cognitive features characteristic of the condition^35–39^.

In this investigation, we aimed to expand upon previous findings of figure disembedding in autism by investigating how autistic and typical individuals make perceptual decisions about two similar or differing figures.

## Methods

### Participants

Participants with normal or corrected-to-normal vision were recruited online via an email notification sent to individuals registered to two University of Cambridge databases: (1) the Autism Research Centre database (accessible at www.autismresearchcentre.com) was used to recruit autistic adults and (2) a second database (accessible at www.cambridgepsychology.com) was used to recruit the non-autistic adult controls. The first database collects information on formal autism diagnoses by asking participants to choose their diagnoses from a drop-down menu. This is followed up by questions about the year of diagnosis, the professional who diagnosed them, and the facility where they were diagnosed. Participants were entered into a prize draw for the chance to win £50. After excluding participants with missing/incomplete data, the dataset contained 140 autistic (82 females) and 147 non-autistic (118 females) adults aged 18–60 years. There were no significance group differences in age (*t*(283) = −0.55, *p* = 0.579) for autism (*Mean* = 35.1, *SD* = 9.85) and controls (*Mean* = 35.8, *SD* = 9.85).

### Procedure

This study was approved by and conducted in accordance with the regulations of the Psychology Research Ethics Committee in Cambridge (PREC. 2015.018). Written informed consent was obtained from all participants. Participants completed behavioural tasks probing working memory and visual perception via *Cambridge Brain Sciences* (www.cambridgebrainsciences.com), a web-based platform for cognitive assessments. Participants were instructed to complete the tasks on a desktop computer while seated comfortably and with a clear view of the screen. Verbal and visuospatial working memory were assessed using the standardised Digit Span test, which measures the ability to recall a sequence of digits, and the Monkey Ladder test, which measures the ability to recall the location of digits^40,41^. Stimuli were scaled to size to account for differences in browsers, devices, and screen size. All tasks were adapted for online computerized testing and validated in large samples^42^.

For each task, participants were given 90 s to complete as many trials as possible, with a timer and the score displayed on one side of the screen. The stimulus presentation was pseudo-randomized such that equal numbers of “match” and “mismatch” trials were administered over 90 s. The difficulty level of each trial increased or decreased based on the participant’s performance on the previous trial. More specifically, there were two trials at each level of difficulty. If the participant got both trials right, the difficulty level increased by one and if they got both wrong, it reduced by one. The following visual scene discrimination tasks were implemented:

### Task 1: Interlocking polygons

**Figure 1:**
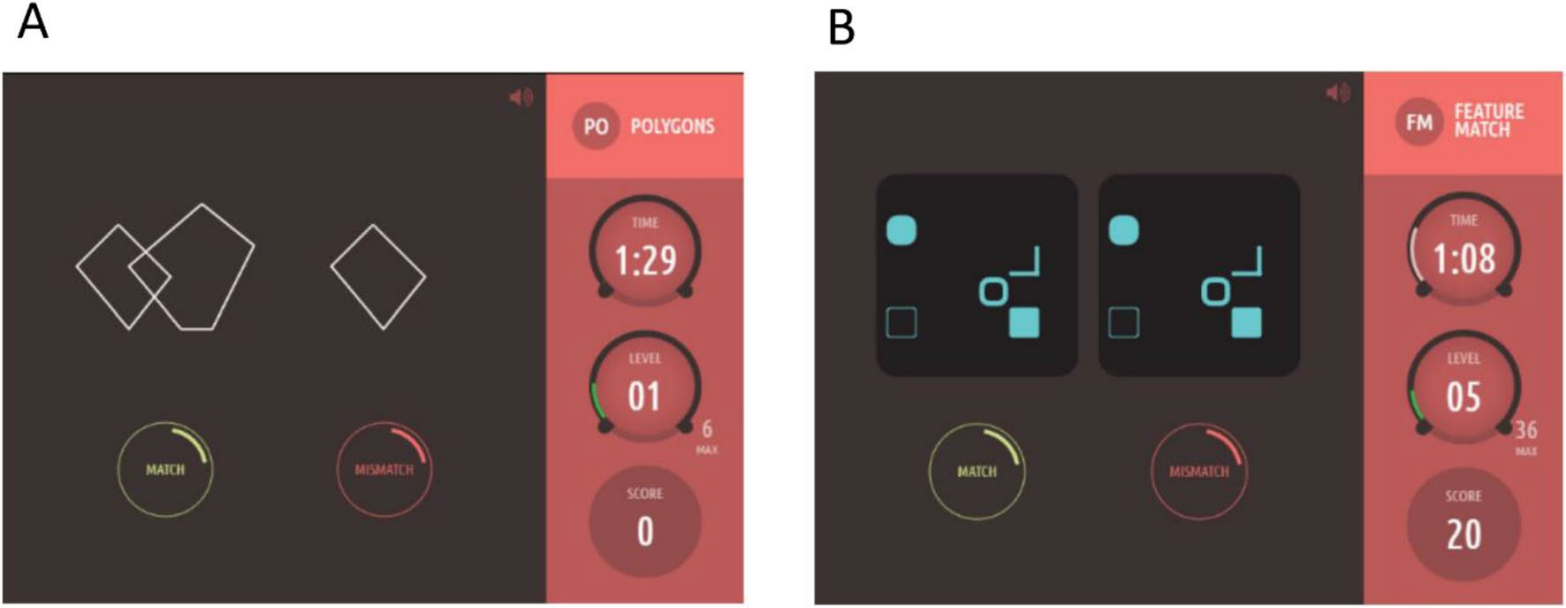
Overview of Cambridge Brain Sciences visual perception tasks. (**A**) Task 1: Interlocking polygons. (**B**) Task 2: Feature match. Participants were instructed to indicate whether a scene displayed on the other side of the screen is identical (“match”) or not identical (“mismatch”) to the other scene. Participants were given 90 s to complete as many trials as possible, with a timer and the score displayed on one side of the screen.

The Interlocking Polygons task is based on pen-and-paper tasks used in clinical neuropsychological tests^43^. In this task, a pair of interlocked polygons is displayed on one side of the screen. Participants were instructed to indicate whether a polygon displayed on the other side of the screen is identical (“match”) or not identical (“mismatch”) to one of the interlocking polygons (Fig. 1A). Difficulty on each trial corresponded to more subtle differences in the polygons.

### Task 2: Feature match

The Feature Match task is a visual search task based on the feature integration theory of visual attention^5^. Arrays of abstract shapes were displayed on either side of the screen. Participants were instructed to indicate whether the arrays’ contents were identical (“match”) or differed by a single shape (“mismatch”) (Fig. 1B). Difficulty on each trial corresponded to an increase in the number of shapes in the array.

### Data analysis

Data were analysed in *R version 4.0.3* (R Core Team, 2020) and *RStudio* (RStudio Team, 2020) with the help of the “*tidyverse”* package^44^. For Bayesian statistics, we used the “*Bayes Factor”* R package and report Bayes factors (BF) which quantify the strength of evidence for the alternative hypothesis (BF_10_) over the null (BF_01_)^45–47^. The magnitude of this strength increases with deviation from 1, with BF_10_ > 3 considered as moderate evidence and BF_10_ > 10 as strong evidence for the alternative hypothesis, while BF_10_ < 3 is insufficient evidence for or against the alternative hypothesis^48–50^. For t-tests, we report t-statistics, p-values, 95% confidence interval (CI) values, and effect sizes in addition to the Bayes factors. The R package *“psycho”* was used for the signal detection theory analyses^51^.

To help address the heterogeneity within our online sample, we first excluded participants whose working memory scores were less than 2 standard deviations from the overall mean. We then conducted exploratory t-tests to measure the extent to which the Autism and Control groups differed in working memory abilities.

As accuracy rates do not adequately capture the participants’ decision criteria, we employed a Signal Detection Theory (SDT) approach to examine the response biases/strategies used by the groups^52,53^. In this approach, we considered the “mismatch” trials as the signal and the “match” trials as noise.

We calculated the sensitivity/discriminability index (d′) of signal from noise using the following formula:

1 $${d}^{{\prime}}=Z\left(hit \,rate\right)-Z (false \,alarm \,rate)$$

The response criterion (C) which measures participant bias in choosing one response was calculated using the following:

2 $$C=\frac{-\left[Z\left(hit\, rate\right)+Z\left(false \,alarm\, rate\right)\right]}{2}$$

where hits and false alarms are expressed as the proportion of responses in each category, and Z(.) is the inverse of the cumulative distribution function of the given Gaussian distribution. We then assessed group differences in sensitivity indices (d´) and response criteria (C) by means of t-tests. Additional analyses of group differences in accuracy rates on each condition are reported in the Supplementary Material.

## Results

### Working memory

After excluding participants whose working memory performance was below the cut-off, 276 participants remained: 129 Autism (75 female, 54 male) and 147 Control (118 female, 29 male). The exploratory t-test on verbal working memory as assessed by the Digit Span test showed evidence in favour of group differences (*BF*_*10*_ = 27, *t*(273) = 3.40, *p* < 0.001, *d* = 0.40, 95% *CI* [0.14, 0.56]) between the Autism (*Mean* = 5.44, *SD* = 0.82) and Control (*Mean* = 5.84, *SD* = 0.91) groups. Meanwhile, between-group results for the visuospatial working memory test yielded a *BF*_*10*_ smaller than 1 (*BF*_10_ = 0.69), with evidence leaning towards a lack of group differences (*t*(273) = 1.87, *p* = 0.06, *d* = 0.22, 95% *CI* [−0.006, 0.27]) between the Autism (*Mean* = 5.07, *SD* = 0.57) and Control (*Mean* = 5.21, *SD* = 0.62) groups. The distribution of working memory scores can be seen in Supplementary Figs. 1 & 2.

### Task 1: Interlocking polygons

**Figure 2:**
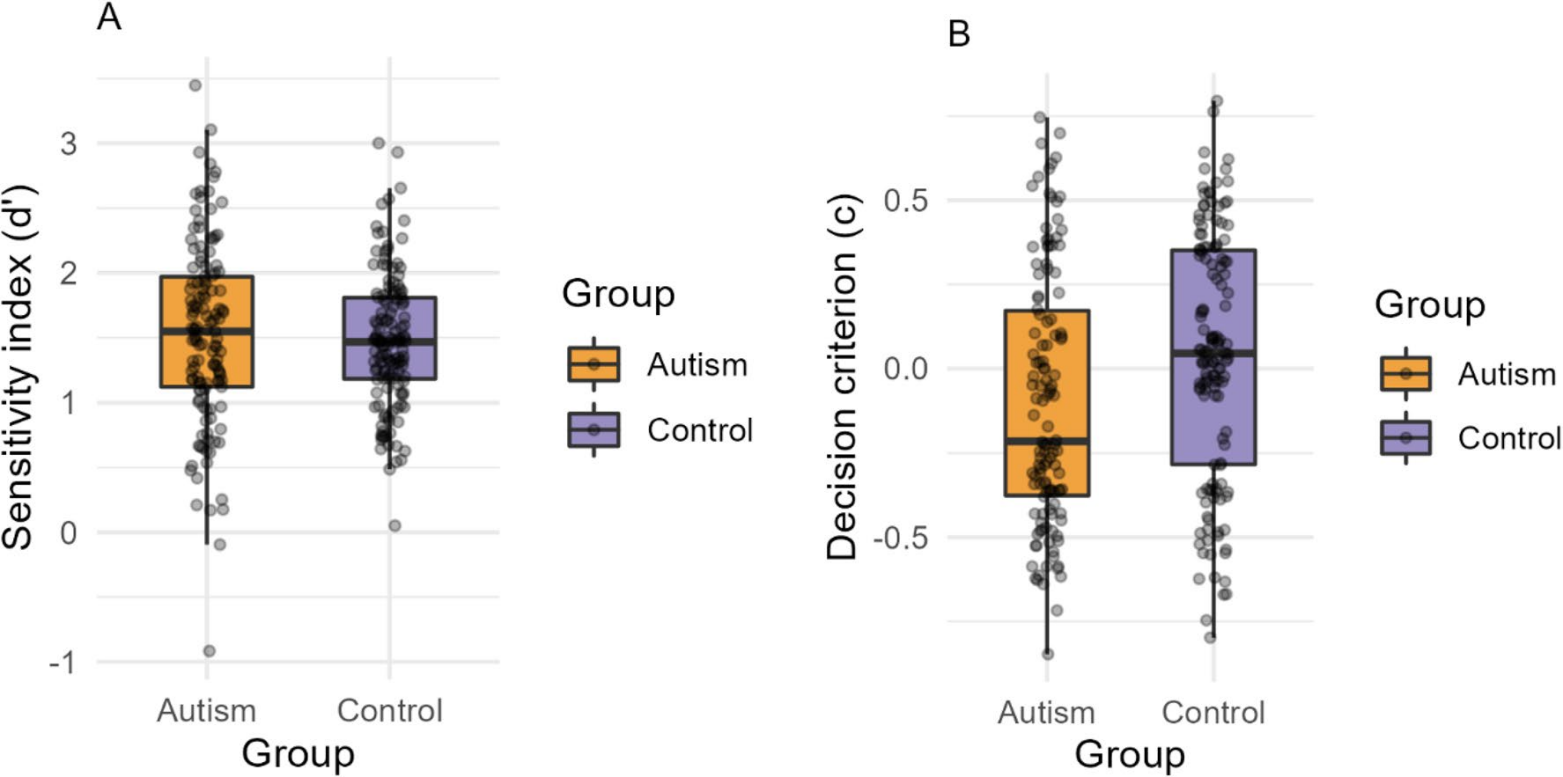
Task 1: Interlocking polygons. (**A**) Signal detection sensitivity index for autism and control groups. (**B**) Signal detection decision criterion values for autism and control groups. Autism group is displayed in orange and control in purple. Dots indicate individual participant results. Error bars show the standard error of the mean.

The independent samples t-test on the total number of trials attempted by each group yielded *BF*_*10*_ = 1.44, suggesting no evidence in favour of group differences (*t*(540) = 2.36, *p* = 0.018, *d* = 0.20, 95% *CI* [0.19, 2.1]) between the Autism (*Mean* = 26.65, *SD* = 3.63) and Control (*Mean* = 25.51, *SD* = 3.60) groups. The mean number of trials completed by both groups in each condition are reported in Supplementary Table 1. The SDT analyses showed no evidence of group differences in the sensitivity index (d′) (*BF*_*10*_ = 0.16, *t*(232) = −0.68, *p* = 0.49, *d* = 0.08, *95%* CI [−0.20, 0.09]) between Autism (*Mean* = 1.52, *SD* = 0.55) and Control (*Mean* = 1.4, *SD* = 0.61) (Fig. 2A). At the same time, we found moderate evidence of group differences in the decision criterion (C) (*BF*_*10*_ = 1.55, *t*(265) = 2.38, *p* = 0.02, *d* = 0.27, *95%* CI [0.22, 0.30]) used by Autism (*Mean* = −0.14, *SD* = 0.55) and Control (*Mean* = 0.02, *SD* = 0.61) groups (Fig. 2B). This suggests a response bias by the Autism group in choosing the “mismatch” option when uncertain, thus leading to more false alarms on the “match” trials (Fig. 2B).

### Task 2: Feature match

**Figure 3:**
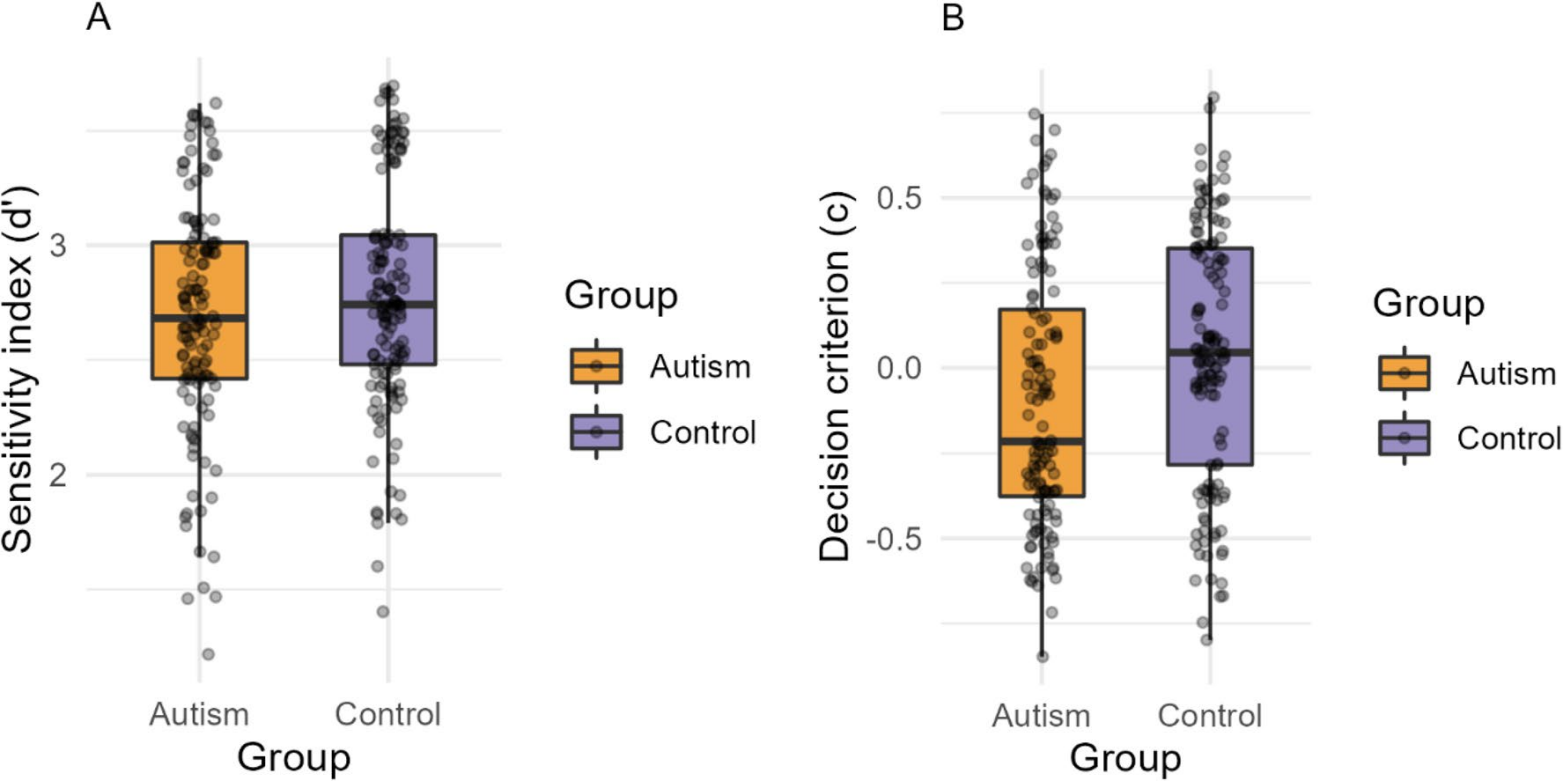
Task 2: Feature match. (**A**) Signal detection sensitivity index for autism and control groups. (**B**) Signal detection decision criterion values for autism and control groups. Autism group displayed in orange and control in purple. Dots indicate individual participant results. Error bars show the standard error of the mean.

The independent samples t-test on the total number of trials attempted by each group yielded *BF*_*10*_ < 1 (*BF*_*10*_ = 0.09) suggesting no evidence of group differences (*t*(539) = 0.22, *p* = 0.82, *d* = 0.02, 95% *CI* [−0.53, 0.67]) between the Autism (*Mean* = 25.5, *SD* = 3.63) and Control (*Mean* = 25.4, *SD* = 3.60) groups (Supplementary Table 1). The SDT analyses showed no evidence of group differences in the sensitivity index (d′) (*BF*_*10*_ = 0.45, *t*(258) = 1.60, *p* = 0.10, *d* = 0.19, *95%* CI [−0.02, 0.22]) between Autism (*Mean* = 2.69, *SD* = 0.50) and Control (Mean = 2.79, *SD* = 0.49) (Fig. 3A). At the same time, we found substantial evidence of group differences in the decision criterion (C) (*BF*_*10*_ = 8.01, *t*(258) = 2.94, *p* = 0.003, *d* = 0.36, *95%* CI [0.04, 0.22]) used by Autism (*Mean* = −0.10, *SD* = *0.37*) and Control (*Mean* = 0.03, *SD* = 0.36) groups (Fig. 3B). This suggests more false alarms by the autism group during “match” trials.

## Discussion

Using a large sample of autistic and typical participants, we conducted two visual cognition tasks to test figure discrimination ability and perceptual decision-making. In Task 1: Interlocking Polygons, participants indicated whether a target polygon was present in the comparison figure of interlocking polygons. In Task 2: Feature Match, participants indicated whether two arrays of shapes differed by a single item. Investigations using Signal Detection Theory indicated no group differences in visual perceptual sensitivity. At the same time, we found group differences in the decision criterion used by the groups. Specifically, autistic participants on average tended to choose the “mismatch” option when faced with uncertainty during “match” trials.

We found no group differences in the sensitivity index (d′), suggesting no clear differences in visual perception between the groups (Figs. 2A, 3A). This contradicts our initial hypotheses of differential visual perception in autism. At the same time, we found a notable response bias by the autism group while making decisions. More specifically, autistic participants were more likely to choose the “mismatch” option, thus leading to more hits on the “mismatch” condition, but also more false alarms on the “match” condition (Figs. 2B, 3B). To measure the extent of this response bias, additional one-sample t-tests on the response criterion (C) were computed separately for each group, which indicated that the autism group tended to choose “mismatch” more often than the ideal observer (Supplementary Material). It has been hypothesized that the perceptual features of autism may be due to variable levels of internal noise, thus leading to difficulties in signal-to-noise separation^39,54–59^. Internal noise in autism has been attributed to atypical neural connectivity of sensory brain networks^35,37,60,61^ and an imbalance in excitatory and inhibitory neurotransmitters^38,62–64^. However, in this study, we found no differences between autistic and control groups in their discriminability indices in both tasks (Figs. 2A, 3A). While our findings indicate group differences in the decision criteria used by the groups (Figs. 2B, 3B), it is unclear *why* autistic individuals use a lower decision criterion while choosing between signal and noise responses. Future research using computational models and neuroimaging methods may shed more light on these findings.

Our study has its limitations: the less-controlled nature of the online task set-up, the sampling bias of participants with access to computers and internet, and the unbalanced sex ratio within our study sample. A greater percentage of female participants reflects what is the norm with online research^65^. However, due to possible sex differences in autism and visual cognition, we acknowledge this as an important caveat. Our findings may be more generalizable to females, however it is ultimately unclear whether the same effects would be observed in a sex-balanced or male-only sample. Future research taking these caveats into account may answer more questions about perceptual inference and decision-making in autism.

In conclusion, contrary to findings from previous research, when compared to typical people, autistic individuals show no differences in visual perceptual sensitivity on two variations of figure disembedding tasks. On the other hand, autistic individuals show a response bias when faced with uncertainty during these tasks, suggesting differences in perceptual decision-making. Taken together, our findings shed light on how autistic individuals make perceptual choices on similarity judgement tasks and provide clear directions for future research.

## Supplementary Information

Supplementary Information.

## Data Availability

The raw datasets generated and/or analysed during the current study are not publicly available as volunteers in the Cambridge Autism Research Database (CARD) did not consent for their data to be deposited in an Open Access archive. However, the CARD Management Committee considers requests by researchers for specific parts of the database (in anonymised form) to test specific hypotheses (please contact: research@autismresearchcentre.com).

## Code availability

All the analyses scripts are publicly shared and can be accessed here: https://github.com/naziajassim/pdm_autism.

## References

[1] Wolfe JM (2021). Guided Search 6.0: An updated model of visual search. Psychon. Bull. Rev..

[2] Bar M (2004). Visual objects in context. Nat. Rev. Neurosci..

[3] Series P, Seitz A (2013). Learning what to expect (in visual perception). Front. Hum. Neurosci..

[4] Summerfield C, Egner T (2009). Expectation (and attention) in visual cognition. Trends Cogn. Sci..

[5] Treisman AM, Gelade G (1980). A feature-integration theory of attention. Cogn. Psychol..

[6] Verghese P (2001). Visual search and attention: A signal detection theory approach. Neuron.

[7] Krantz DH (1969). Threshold theories of signal detection. Psychol. Rev..

[8] Summerfield C, Blangero A, Dreher JC, Tremblay L (2017). Chapter 12-Perceptual decision-making: What do we know, and what do we not know?. Decision Neuroscience.

[9] Wyart V, Nobre AC, Summerfield C (2012). Dissociable prior influences of signal probability and relevance on visual contrast sensitivity. Proc. Natl. Acad. Sci..

[10] Bergmann N, Koch D, Schubö A (2019). Reward expectation facilitates context learning and attentional guidance in visual search. J. Vis..

[11] Luria R, Vogel EK (2011). Visual search demands dictate reliance on working memory storage. J. Neurosci..

[12] Wolfe JM, Horowitz TS (2017). Five factors that guide attention in visual search. Nat. Hum. Behav..

[13] Baron-Cohen, S. *The Pattern Seekers: A New Theory of Human Invention*. Penguin UK; 2020.

[14] O’Riordan MA, Plaisted KC, Driver J, Baron-Cohen S (2001). Superior visual search in autism. J. Exp. Psychol. Hum. Percept. Perform..

[15] Robertson CE, Kravitz DJ, Freyberg J, Baron-Cohen S, Baker CI (2013). Tunnel vision: Sharper gradient of spatial attention in autism. J. Neurosci..

[16] Robertson CE, Baron-Cohen S (2017). Sensory perception in autism. Nat. Rev. Neurosci..

[17] Kenny L, Hattersley C, Molins B, Buckley C, Povey C, Pellicano E (2016). Which terms should be used to describe autism? Perspectives from the UK autism community. Autism.

[18] American Psychiatric Association. Diagnostic and Statistical Manual of Mental Disorders [Internet]. Fifth Edition. American Psychiatric Association; 2013 [cited 2020 Mar 24]. 10.1176/appi.books.9780890425596

[19] Baron-Cohen S, Ashwin E, Ashwin C, Tavassoli T, Chakrabarti B (2009). Talent in autism: Hyper-systemizing, hyper-attention to detail and sensory hypersensitivity. Philos. Trans. R. Soc. B Biol. Sci..

[20] Happé F, Frith U (2006). The weak coherence account: Detail-focused cognitive style in autism spectrum disorders. J. Autism Dev. Disord..

[21] Mottron L, Dawson M, Soulières I, Hubert B, Burack J (2006). Enhanced perceptual functioning in autism: An update, and eight principles of autistic perception. J. Autism Dev. Disord..

[22] Shah A, Frith U (1983). An islet of ability in autistic children: A research note. J. Child Psychol. Psychiatry.

[23] Jolliffe T, Baron-Cohen S (1997). Are people with autism and asperger syndrome faster than normal on the embedded figures test?. J. Child Psychol. Psychiatry.

[24] Plaisted K, O’Riordan M, Baron-Cohen S (1998). Enhanced visual search for a conjunctive target in autism: A research note. J. Child Psychol. Psychiatry.

[25] Almeida RA, Dickinson JE, Maybery MT, Badcock JC, Badcock DR (2010). A new step towards understanding Embedded Figures Test performance in the autism spectrum: The radial frequency search task. Neuropsychologia.

[26] Brian JA, Bryson SE (1996). Disembedding performance and recognition memory in autism/PDD. J. Child Psychol. Psychiatry.

[27] Edgin JO, Pennington BF (2005). Spatial cognition in autism spectrum disorders: Superior, impaired, or just intact?. J. Autism Dev. Disord..

[28] White SJ, Saldaña D (2011). Performance of children with autism on the embedded figures test: A closer look at a popular task. J. Autism Dev. Disord..

[29] Chang DHF, Kourtzi Z, Welchman AE (2013). Mechanisms for extracting a signal from noise as revealed through the specificity and generality of task training. J. Neurosci..

[30] Dosher BA, Lu ZL (1998). Perceptual learning reflects external noise filtering and internal noise reduction through channel reweighting. Proc. Natl. Acad. Sci. USA.

[31] Hurlbert A (2000). Visual perception: Learning to see through noise. Curr. Biol..

[32] Levi DM, Klein SA, Chen I (2005). What is the signal in noise?. Vis. Res..

[33] Zanker JM, Braddick OJ (1999). How does noise influence the estimation of speed?. Vis. Res..

[34] Faisal AA, Selen LPJ, Wolpert DM (2008). Noise in the nervous system. Nat. Rev. Neurosci..

[35] Belmonte MK, Allen G, Beckel-Mitchener A, Boulanger LM, Carper RA, Webb SJ (2004). Autism and abnormal development of brain connectivity. J. Neurosci..

[36] Dakin S, Frith U (2005). Vagaries of visual perception in autism. Neuron.

[37] Dinstein I, Heeger DJ, Lorenzi L, Minshew NJ, Malach R, Behrmann M (2012). Unreliable evoked responses in autism. Neuron.

[38] Rubenstein JLR, Merzenich MM (2003). Model of autism: Increased ratio of excitation/inhibition in key neural systems. Genes Brain Behav..

[39] Davis G, Plaisted-Grant K (2015). Low endogenous neural noise in autism. Autism.

[40] Inoue S, Matsuzawa T (2007). Working memory of numerals in chimpanzees. Curr. Biol..

[41] Wechsler D (1981). The psychometric tradition: Developing the Wechsler Adult Intelligence Scale. Contemp. Educ. Psychol..

[42] Hampshire A, Highfield RR, Parkin BL, Owen AM (2012). Fractionating human intelligence. Neuron.

[43] Folstein MF, Folstein SE, McHugh PR (1975). “Mini-mental state”: A practical method for grading the cognitive state of patients for the clinician. J. Psychiatr. Res..

[44] Wickham H, Averick M, Bryan J, Chang W, McGowan LD, François R (2019). Welcome to the tidyverse. J. Open Source Softw..

[45] Jarosz AF, Wiley J (2014). What are the odds? A practical guide to computing and reporting Bayes factors. J. Probl. Solving..

[46] Morey, R. D., Rouder, J. N., Jamil, T., Urbanek, S., Forner, K., & Ly, A. BayesFactor: Computation of Bayes Factors for Common Designs [Internet]. 2021 [cited 2022 Feb 3]. Available from: https://CRAN.R-project.org/package=BayesFactor

[47] Rouder JN, Morey RD, Speckman PL, Province JM (2012). Default Bayes factors for ANOVA designs. J. Math. Psychol..

[48] Keysers C, Gazzola V, Wagenmakers EJ (2020). Using Bayes factor hypothesis testing in neuroscience to establish evidence of absence. Nat. Neurosci..

[49] Lee MD, Wagenmakers EJ (2014). Bayesian Cognitive Modeling: A Practical Course.

[50] Ly A, Verhagen J, Wagenmakers EJ (2016). Harold Jeffreys’s default Bayes factor hypothesis tests: Explanation, extension, and application in psychology. J. Math. Psychol..

[51] Makowski D (2018). The psycho package: An efficient and publishing-oriented workflow for psychological science. J. Open Source Softw..

[52] Harvey, L. O., Hammond, K. R., Lusk, C., & Mross, E. F. The Application of Signal Detection Theory to Weather Forecasting Behavior. 1992.

[53] Stanislaw H, Todorov N (1999). Calculation of signal detection theory measures. Behav. Res. Methods Instrum. Comput..

[54] Franklin A, Sowden P, Notman L, Gonzalez-Dixon M, West D, Alexander I (2010). Reduced chromatic discrimination in children with autism spectrum disorders. Dev. Sci..

[55] Park WJ, Schauder KB, Zhang R, Bennetto L, Tadin D (2017). High internal noise and poor external noise filtering characterize perception in autism spectrum disorder. Sci. Rep..

[56] Simmons DR, Robertson AE, McKay LS, Toal E, McAleer P, Pollick FE (2009). Vision in autism spectrum disorders. Vis. Res..

[57] Torres EB, Denisova K (2016). Motor noise is rich signal in autism research and pharmacological treatments. Sci. Rep..

[58] Van de Cruys S, Evers K, Van der Hallen R, Van Eylen L, Boets B, de Wit L (2014). Precise minds in uncertain worlds: Predictive coding in autism. Psychol. Rev..

[59] Van de Cruys S, Van der Hallen R, Wagemans J (2017). Disentangling signal and noise in autism spectrum disorder. Brain Cogn..

[60] Jassim, N., Baron-Cohen, S., & Suckling, J. Meta-analytic evidence of differential prefrontal and early sensory cortex activity during non-social sensory perception in autism. *Neurosci. Biobehav. Rev.* [Internet]. 2021 Apr 19 [cited 2021 Apr 20]; Available from: https://www.sciencedirect.com/science/article/pii/S0149763421001664 10.1016/j.neubiorev.2021.04.014 33887326

[61] Simmons DR (2019). Some clarifications on neural noise and sensory sensitivities in Autism. Cogn. Neurosci..

[62] Gogolla N, LeBlanc JJ, Quast KB, Südhof TC, Fagiolini M, Hensch TK (2009). Common circuit defect of excitatory–inhibitory balance in mouse models of autism. J. Neurodev. Disord..

[63] Kurcyus K, Annac E, Hanning NM, Harris AD, Oeltzschner G, Edden R (2018). Opposite dynamics of GABA and glutamate levels in the occipital cortex during visual processing. J. Neurosci..

[64] Robertson CE, Ratai EM, Kanwisher N (2016). Reduced GABAergic action in the autistic brain. Curr. Biol..

[65] Smith, W. Does gender influence online survey participation? A record-linkage analysis of university faculty online survey response behavior. Online Submission. (2008).

