# Supplementary material for: Perceptual decision-making in autism as assessed by “spot the difference” visual cognition tasks
Source: Sci Rep. 2022 Sep 14;12:15458. doi: 10.1038/s41598-022-19640-4 (PMC9474452; doi:10.1038/s41598-022-19640-4)
Supplement: Supplementary file 1 — Supplementary Information. [file 41598_2022_19640_MOESM1_ESM.docx]

Supplementary Material

1. Supplementary Table 1: Summary statistics from Task 1: Interlocking Polygons and Task 2: Feature Match

| Task | Group | Task Condition | Number of Trials Attempted  *Mean (SD)* | Accuracy Rate  *Mean (SD)* |
| --- | --- | --- | --- | --- |
| 1.  Interlocking Polygons | Autism | Match | 12.7 (2.9) | 71.1 (20.2) |
|  |  | Mismatch | 12.7 (2.4) | 79.7 (20.5) |
|  | Control | Match | 13.2 (3.2) | 76.2 (21.6) |
|  |  | Mismatch | 12.4 (2.1) | 73.1 (20) |
| 2.  Feature Match | Autism | Match | 12.8 (2.1) | 89.8 (10.3) |
|  |  | Mismatch | 12.7 (2.4) | 93.4 (9.8) |
|  | Control | Match | 13 (2.4) | 93.4 (9.1) |
|  |  | Mismatch | 12.5 (2.1) | 92.6 (9.4) |

2) Working Memory scores:


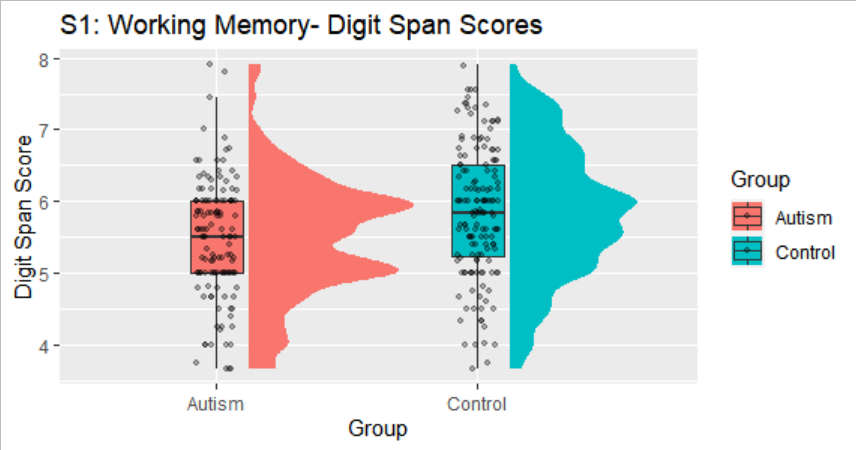


Fig S1. Digit Span Task: Distribution of scores for Autism (pink) and Control (blue). Dots indicate individual data points. Circles indicate outliers. Error bars show the standard error of the mean.


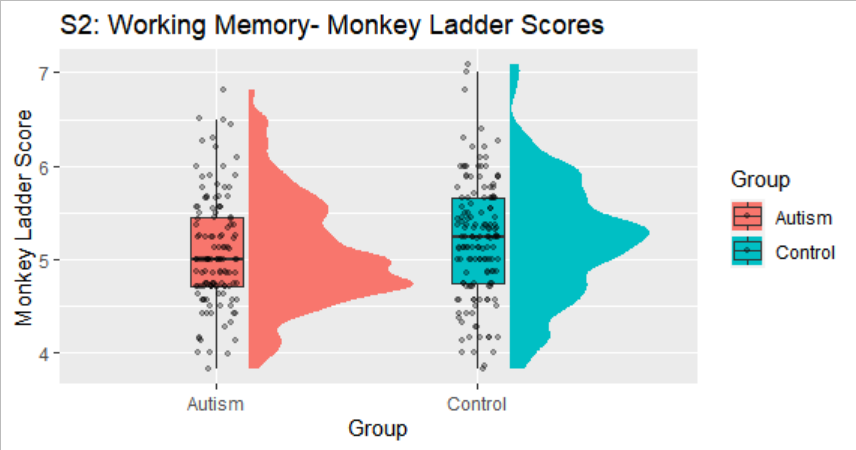


Fig S2. Monkey Ladder Task: Distribution of scores for Autism (pink) and Control (blue). Dots indicate individual data points. Circles indicate outliers. Error bars show the standard error of the mean

3) Accuracy rates:

For both tasks, overall and condition-specific accuracy rates were computed for each group. We first conducted independent two-sample t-tests on the total number of trials attempted by each group to ensure that accuracy rate group differences could not be attributed to differences in this measure. To investigate differences in task performance due to task condition and group, a Bayesian 2x2 factorial Analysis of Variance (ANOVA) was computed on accuracy rates with group (Autism vs Control) and task condition (Match vs Mismatch) as factors. In line with the requirements of Bayesian ANOVA, each individual participant was included as a random factor.

Task 1 Interlocking Polygons: The Bayesian ANOVA on accuracy rates with group and task condition as factors showed evidence against the main effects of group (*BF_10_* = 0.10) and task condition (*BF_10_* = 0.22).  Meanwhile, we found strong positive evidence in favour of an interaction effect of group and task condition (*BF_10_* =28), with the Autism group making more accurate responses in the “mismatch” condition and faring worse on the “match” condition (*Supplementary Fig S3*).

Task 2 Feature Match: A ceiling effect was observed in the mean accuracy rates across both the Autism (*Mean*=91.6, *SD*=6.35) and Control (*Mean*=93, *SD*=5.56) groups, both overall and when split according to condition *(Supplementary Fig S4, Supplementary Table 1)*.The Bayesian ANOVA on accuracy rates with group and task condition as factors showed evidence against the main effect of group (*BF_10_*=0.31) and task condition (*BF_10_*=0.16). Meanwhile, there was strong evidence in favour of an interaction effect of group and task condition (*BF_10_*=42) , with the Autism group making more accurate responses on the “mismatch” condition *(Supplementary Fig S4)*.


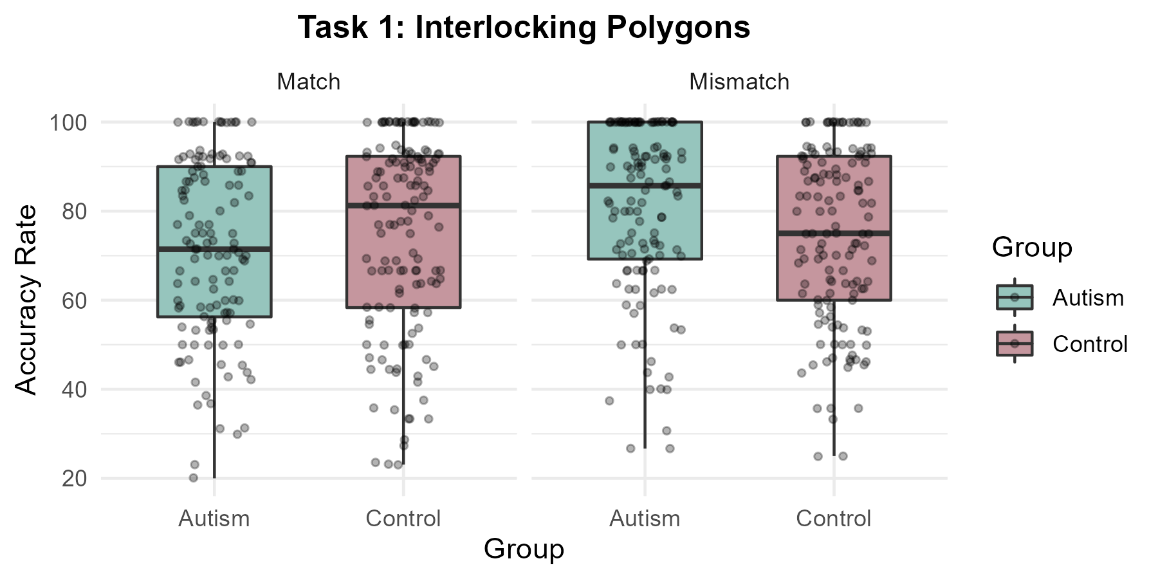


Fig S3: Task 1: Interlocking Polygons. Mean accuracy rates for autism (in teal) and control (pink), split by condition. Autism group is displayed in teal and control in pink. Dots indicate individual participant results. Error bars show the standard error of the mean.


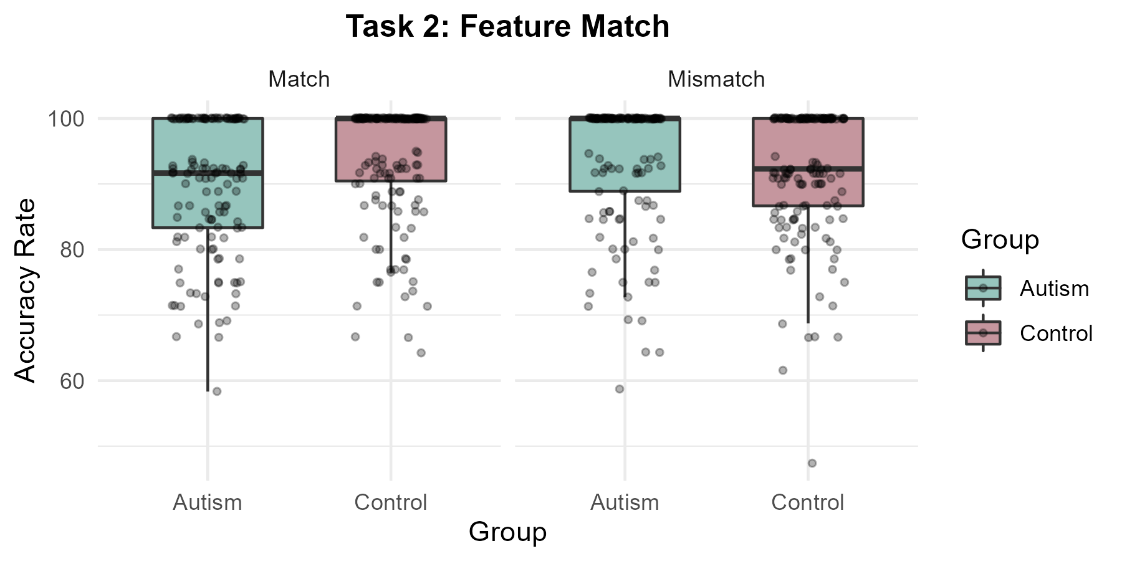


Fig S4: Task 2: Feature Match. Mean accuracy rates for autism (in teal) and control (pink), split by condition. Autism group is displayed in teal and control in pink. Dots indicate individual participant results. Error bars show the standard error of the mean.

4) One-sample t-tests:

We conducted one-sample t-tests separately for each group to investigate whether the decision criterion ‘c’ significantly differed from 1

1. Interlocking Polygons: For the autism group, the decision criteria (c) was significantly lower than 1: t(127) = -3.02, p =0.003, d = -0.267. Meanwhile, for the control group, the decision criteria (c) did not significantly differ from 1: t(146) = 1.07, p =0.28, d = 0.008.

2. Feature Match: For the autism group, the decision criteria (c) was significantly lower than 1: t(124) = -3.5, p =0.0028, d = -0.272. Meanwhile, for the control group, the decision criteria (c) did not significantly differ from 1: t(145) = 1.57, p =0.12, d = 0.130.
